# Prevalence of melasma in pregnant women and its relationship with body perception: a cross-sectional study in Türkiye

**DOI:** 10.1590/1516-3180.2026.3641.27032026

**Published:** 2026-07-17

**Authors:** Demet Toktaş, Rabia Atilla

**Affiliations:** I Midwife, Niğde Ömer Halisdemir University Training and Research Hospital, Niğde, Türkiye.; II Assistant Professor, Department of Nursing, Zübeyde Hanım Faculty of Health Sciences, Niğde Ömer Halisdemir University, Niğde, Türkiye.

**Keywords:** Pregnancy, Melanosis, Chloasma, Body image, Perception, Prevalence, Risk factors, Melasma, Body perception, Nursing

## Abstract

**BACKGROUND:**

Melasma is one of the most common physiological hyperpigmentation disorders observed during pregnancy, and its clinical characteristics and risk factors have been extensively investigated. However, evidence regarding the relationship between pregnancy-related melasma and body perception remains limited.

**OBJECTIVE:**

This descriptive cross-sectional study aimed to determine the prevalence of melasma among pregnant women and to examine its association with body perception.

**METHODS:**

This study included 450 pregnant women in the central city center of the Central Anatolia Region of Türkiye who met the inclusion criteria. Data were collected using the Introductory Information Form, the Melasma and Skin Type Characteristics Assessment Form, and the Body Perception Scale (BPS) and analyzed using descriptive statistics, comparative analyses, and logistic regression.

**RESULTS:**

Melasma was identified in 37.8% of the participants, and the centrofacial pattern was the most common presentation (56.5%). A statistically significant association was found between pregnancy-related melasma and total BPS scores (*p* < 0.05). Pregnant women without melasma had significantly higher mean BPS scores than those with melasma (150.83 ± 17.18 vs. 146.15 ± 15.16; *p* = 0.004). Logistic regression analysis demonstrated that low educational and income levels, a personal or family history of melasma, and Fitzpatrick skin type IV were significant predictors of pregnancy-related melasma (*p* < 0.05), accounting for 61% of the variance.

**CONCLUSION:**

The findings indicate the need for a holistic assessment of physiological skin changes and body perception during pregnancy, as well as the integration of counseling, education, and nursing care interventions targeting risk factors into routine prenatal care.

## INTRODUCTION

Melasma is an acquired, chronic hyperpigmentation disorder characterized by increased melanin deposition in the epidermal and/or dermal layers of the skin.^1^ Clinically, it presents as asymptomatic light-to-dark-brown macules with irregular borders and symmetrical distribution, most commonly affecting sun-exposed areas, such as the face and neck.^1,2^ Although the exact etiology remains unclear, genetic predisposition, environmental exposure, and hormonal changes are known to contribute to its development. Pregnancy is a major risk factor, largely because of increased levels of estrogen, progesterone, and melanocyte-stimulating hormone.^2,3^ The prevalence of melasma during pregnancy has been reported to range from 36.4% to 75%, varying by geographic region and skin type.^3^ Although spontaneous regression is generally expected within one year postpartum, approximately 30% of cases persist.^4^ Although considered a physiological change, pregnancy-related melasma may lead to aesthetic concerns and negatively affect body image perception among pregnant women.^5,6^

Body perception refers to an individual's perception of their physical appearance, along with associated feelings and thoughts.^7^ Physiological and psychological changes during pregnancy influence body perceptions of the expectant mother in multiple ways^8^ and in some cases, may contribute to poor body image.^9^ During pregnancy, discrepancies between changes in a woman's face and body and her internalized ideal body image may result in either positive or negative body image perceptions.^6^ Visible skin changes, particularly pregnancy mask (melasma), striae gravidarum, and edema, have been associated with increased body dissatisfaction.^5^ Qualitative studies have shown that many pregnant women perceive pregnancy-related melasma as distressing and upsetting, report feeling unattractive, limit social interactions, and experience anxiety about whether their skin will return to its pre-pregnancy state.^6,10^

Healthcare professionals play a significant role in identifying aesthetic concerns related to pregnancy-associated melasma and planning preventive and treatment-related care.^4,11,12^ However, given the limited number of studies addressing this topic, there is a need for a comprehensive examination of the prevalence of pregnancy-related melasma and its association with body perception.

## OBJECTIVES

This study aimed to determine the prevalence of melasma among pregnant women and to examine its relationship with body perception.

## METHODS

### Study design and population

This study employed descriptive cross-sectional design. It was conducted between March and May 2024 in a city in the Central Anatolia Region of Türkiye.

The study population comprised pregnant women in their second or third trimester who attended routine examinations at the obstetrics and gynecology outpatient clinic of a training and research hospital in the Central Anatolia Region of Türkiye. The inclusion criteria were as follows: pregnant women aged ≥18 years who were in the second or third trimester, able to communicate in Turkish, and voluntarily agreed to participate. Pregnant women in the first trimester; those with multiple pregnancies; those diagnosed during pregnancy with dermatological conditions (e.g., psoriasis, atopic dermatitis, urticaria, connective tissue diseases, skin cancer, and pemphigus); those with pregnancy-related dermatoses, including hemangioma, cholestasis, spider angioma, palmar erythema, and malignant melanoma; those with facial manifestations of these conditions; and those with a diagnosed psychiatric disorder were excluded.

The sample size was calculated using the formula for estimating sample size in populations of unknown size. Based on an assumed melasma prevalence of 23.5%,^12^ the minimum required sample size was calculated to be 278.^13^ Participants were recruited between March 1 and May 15, 2024, using convenience sampling. The study was completed with the participation of a total of 450 pregnant women who met the inclusion criteria during the data collection process and consented to participate.

### Measures

Introductory information form: This form was developed by the researchers based on a review of relevant literature^2,4,5,12,14,15^ and expert opinions. It comprised 21 items assessing participants’ sociodemographic and obstetric characteristics.

Melasma and skin type characteristics assessment form: This form comprised nine items assessing participants’ skin type, presence of melasma, personal and family history of melasma, use of preventive measures, and health-care-seeking behaviors, including physician consultation and receipt of treatment.^2,4,5,12,14^ The findings obtained through the physical examination conducted by the researcher—including the pregnant women's natural hair color, eye color, skin tone, presence and number of facial freckles/moles, pregnancy melasma status, melasma type, and skin type characteristics—were evaluated and recorded using this form. Skin type was additionally assessed by the researchers through physical examination according to the Fitzpatrick skin type classification,^16^ and the findings were recorded on the form.

Body perception scale (BPS): The BPS is a 40-item instrument designed to assess satisfaction with different body regions and bodily functions. In this five-point Likert-type scale, each item is rated from 1 (strongly dislike) to 5 (strongly like). Total scores range from 40 to 200, with higher scores indicating greater satisfaction. In the Turkish reliability study conducted by Hovardaoğlu,^17^ Cronbach's alpha coefficient was 0.91; in the present study, it was 0.896.

### Data collection

Data were collected through face-to-face interviews conducted by researchers between March and May 2024, using the ‘Introductory information form’, ‘Melasma and skin type characteristics assessment form’, and ‘BPS’. Facial regions of the participants were assessed by the researchers during a physical examination. Melasma status and skin type were recorded using relevant assessment forms.

### Statistical analyses

Data were analyzed using IBM SPSS Statistics for Windows (version 25; IBM Corp., Armonk, New York). Descriptive statistics, including frequencies, percentages, means, and standard deviations, were used to summarize the data. Normality of the data distribution was assessed using the Kolmogorov–Smirnov test. As the data were normally distributed, independent-samples *t*-tests were used. Logistic regression analysis was performed to evaluate categorical dependent variables. Variables that were significant in the chi-square analyses were entered into the regression model. Statistical significance was set at *p* < 0.05.

### Ethical considerations

Ethical approval was obtained from the Ethics Committee of the university in the province where the study was conducted (Decision No: 2024/01-65; January 31, 2024). Institutional permission was also obtained, and all procedures were performed in accordance with the principles of the Declaration of Helsinki.

## RESULTS

The distribution of selected descriptive characteristics of the pregnant women is presented in Table 1. Pregnancy-related melasma was identified in 37.8% of the participants, of whom 56.5% exhibited the centrofacial type. Furthermore, 40.2% of all women had Fitzpatrick skin type III. Overall, 31.6% of participants were high school graduates, 79.1% were unemployed, 62.8% resided in urban areas, and 70.9% reported that their income was equal to their expenses. Most of the pregnant women reported no history of melasma prior to pregnancy (90.9%), during a previous pregnancy (72.3%), or in their family (76.9%). The majority (99.3%) reported not using any specific preventive methods for melasma; 46.2% applied sunscreen to the face, 21.1% reported consistently applying sunscreen, and 88.2% did not reapply it during the day.

**Table 1:** Distribution of selected descriptive characteristics of the pregnant women (n = 450)

Characteristics		$\bar{\text{X}}$ ± SD	Min–Max/Range
Age (years)		27.23 ± 5.29	18-44 (26)
Gravidity		2.2 ± 1.33	1–8 (7)
Gestational age (weeks)		33.63 ± 4.75	16–41 (25)
Pregnancy BMI (kg/m²)		29.62 ± 4.55	19.1–44.4 (25.3)
Gestational weight gain (kg)		11.11 ± 5.16	0–26 (26)
		**n**	**%**
Presence of pregnancy-related melasma	Present	170	37.8
	Not present	280	62.2
Type of pregnancy melasma (n = 170)	Centrofacial type	96	56.4
	Malar type	70	41.2
	Mandibular type	4	2.4
Fitzpatrick skin type*	Skin type II	95	21.1
	Skin type III	181	40.2
	Skin type IV	174	38.7
Educational level	Literate/primary school	38	8.4
	Secondary school	105	23.3
	High school	142	31.6
	University	133	29.6
	Postgraduate	32	7.1
Employment status	Employed	94	20.9
	Unemployed	356	79.1
Place of residence	Province	283	62.8
	District	70	15.6
	Village	97	21.6
History of melasma before pregnancy	Present	41	9.1
	Not present	409	90.9
History of melasma in previous pregnancy (n = 274)	Present	76	27.7
	Not present	198	72.3
Family history of melasma	Present	104	23.1
	Not present	346	76.9
Use of preventive methods for melasma	Yes	3	0.7
	No	447	99.3
Use of sunscreen on the face	Yes	208	46.2
	No	242	53.8
Frequency of sunscreen use	Always	95	21.1
	Occasionally	113	25.1
	Never	242	53.8
Reapplication of sunscreen during the day	Yes	53	11.8
	No	397	88.2
Physician consultation for pregnancy melasma	Yes	5	1.1
	No	445	98.9

$\bar{\text{X}}$ , mean; SD, standard deviation; Min–Max, minimum–maximum; n, calculations were based only on respondents; BMI, body mass index.

* There were no participants with skin type I or V in the study.

A comparison of BPS scores according to pregnancy-related melasma status is presented in Table 2. A significant difference was observed between the total BPS scores and the presence of melasma during pregnancy (*p* < 0.05). The mean total BPS score was higher among pregnant women without melasma (150.83 ± 17.18) than among those with melasma (146.15 ± 15.16).

**Table 2:** Comparison of body perception scale scores based on pregnancy-related melasma status

Pregnancy-Related Melasma Status	$\bar{\text{X}}$ ± SD	Min–Max (Range)	t test	*p* value
Present	146.15 ± 15.16	80–195 (115)		
Not present	150.83 ± 17.18	93–199 (106)	−2.924	**0.004** *

SD, standard deviation; Min–Max, minimum–maximum.

* *p* < 0.05;

t test, independent samples *t* test.

The distribution of variables associated with the presence of pregnancy-related melasma is presented in Table 3. Logistic regression analysis showed that educational level, income status, history of melasma prior to pregnancy, history of melasma in previous pregnancies, family history of melasma, and skin type were significantly associated with pregnancy-related melasma (*p* < 0.05). The likelihood of pregnancy-related melasma was 3.32 times lower among women whose income was equal to their expenses than among those whose income was less than their expenses (1/0.301 = 3.32; odds ratio [OR] = 0.301; *p* = 0.019). Compared with women with primary school education or below, the likelihood of pregnancy-related melasma was 5.26 times lower among high school graduates (1/0.19 = 5.26; OR = 0.19; *p* = 0.008) and 7.09 times lower among university graduates (1/0.141 = 7.09; OR = 0.141; *p* = 0.005).

**Table 3:** Distribution of variables associated with the presence of pregnancy-related melasma

		β	SE	Wald	*p* value	OR (B)	95% CI OR	
							Lower	Upper
Educational level								
	*Literate/primary school*			10.082	0.039			
	*Secondary school*	−0.861	0.588	2.139	0.144	0.423	0.133	1.34
	*High school*	−1.662	0.627	7.031	**0.008** *	0.19	0.056	0.648
	*University*	−1.961	0.702	7.811	**0.005** *	0.141	0.036	0.557
	*Postgraduate*	−1.741	0.961	3.283	0.07	0.175	0.027	1.153
Place of residence								
	*Village*			3.011	0.222			
	*Province*	−0.858	0.507	2.866	0.09	0.424	0.157	1.145
	*Village*	−0.296	0.581	0.26	0.61	0.744	0.238	2.323
Social security (None)								
	*Yes*	0.467	0.458	1.041	0.308	1.595	0.65	3.914
Perceived income status								
	*Less than expenses*			6.164	**0.046** *			
	*Equal to expenses*	−1.201	0.51	5.536	**0.019** *	0.301	0.111	0.818
	*Greater than expenses*	−0.519	0.747	0.483	0.487	0.595	0.138	2.572
Number of births								
	*3 or more*			5.443	0.142			
	*No previous births*	0.718	0.818	0.77	0.38	2.049	0.413	10.175
	*1*	0.691	0.651	1.126	0.289	1.995	0.557	7.146
	*2*	−0.282	0.65	0.188	0.665	0.754	0.211	2.697
Hormone therapy for infertility treatment (Not received)								
	*Received*	0.489	0.718	0.464	0.496	1.631	0.399	6.657
History of combined oral contraceptive use (Not used)								
	*Used*	0.094	0.55	0.029	0.864	1.099	0.374	3.227
History of melasma before pregnancy (Not present)								
	*Present*	2.252	0.964	5.461	**0.019** *	9.503	1.438	62.809
History of melasma in previous pregnancy (Present)								
	*Not present*	2.65	0.552	23.016	**0** *	14.16	4.795	41.814
Family history of melasma (Present)								
	*Not Present*	1.335	0.475	7.907	**0.005** *	3.798	1.498	9.629
Frequency of sunscreen application								
	*Never*			3.769	0.152			
	*Always*	−0.579	0.574	1.016	0.313	0.561	0.182	1.727
	*Occasionally*	0.59	0.445	1.764	0.184	1.805	0.755	4.314
Regular medication use (No)								
	*Yes*	-0.364	0.613	0.352	0.553	0.695	0.209	2.311
Fitzpatrick skin type								
	*Skin type II*			25.423	**0** *			
	*Skin type III*	0.831	0.572	2.11	0.146	2.296	0.748	7.045
	*Skin type IV*	2.515	0.582	18.649	**0** *	12.365	3.949	38.715
Model statistics		Hosmer–Lemeshow Test = 11.246, *p* = 0.188; Cox & Snell R²=0.457; Nagelkerke R² = 0.61						

β, Regression coefficient;

* *p* < 0.05;

SE, standard error; OR, odds ratio; CI, confidence interval.

In contrast, the likelihood of pregnancy-related melasma was 12.36 times higher among women with Fitzpatrick skin type IV than among those with skin type II (OR = 12.365; *p* < 0.001). A history of melasma prior to pregnancy was associated with a 9.50-fold increase in the likelihood of pregnancy-related melasma (OR = 9.503; *p* = 0.019), a history of melasma in previous pregnancies with a 14.16-fold increase (OR = 14.16; *p* < 0.001), and a family history of melasma with a 3.80-fold increase (OR = 3.798; *p* = 0.005). The final model accounted for 61% of the variance in the presence of melasma during pregnancy.

## DISCUSSION

This study examined the prevalence of pregnancy-related melasma, its association with body perception, and factors associated with its occurrence among pregnant women. The findings indicate that approximately four out of ten pregnant women experienced pregnancy-related melasma, and the presence of melasma was associated with negative body perception. Furthermore, skin type, history of melasma, and educational and income levels were identified as significant factors that correlated with to its occurrence.

In this study, pregnancy-related melasma was identified in 37.8% of pregnant women based on physical examinations performed by the researchers. Globally, the prevalence of melasma during pregnancy has been reported to range from 36.4% to 75%.^3^ Substantial variations in prevalence rates have been reported across countries. Reported prevalence rates are 10.7% in Brazil,^18^ 15.8% in Iran,^15^ 50.8% in India,^19^ and 63.5% in Pakistan.^20^ In Türkiye, the reported prevalence rates range from 23.5% to 46.9%.^12,21^ Differences in skin type, hormonal changes, and geographic factors may contribute to this variability. Although the prevalence observed in the present study is consistent with the findings from other studies conducted in Türkiye, it can be considered moderate relative to international reports.

Most participants in the present study had a Fitzpatrick skin type of III, and approximately half of the melasma cases exhibited a centrofacial type. These findings are consistent with previous reports indicating that skin type III is common among pregnant women,^14,22,23^ and that the centrofacial pattern is the most frequently observed clinical presentation in pregnant women with melasma.^23,24^ Furthermore, most participants did not use specific preventive measures for melasma. Although nearly half reported applying sunscreen to the face, only one-fifth reported consistent use, and the majority did not reapply sunscreen during the day. Similar patterns of limited sun-protective behavior and irregular use of preventive measures during pregnancy have been reported in previous studies.^22,25^

Melasma, although generally considered a physiological skin change during pregnancy, has been reported to adversely affect body perception.^5^ In this study, it was determined that women with pregnancy-related melasma had significantly lower BPS scores compared to women without melasma; in other words, melasma reduced body perception satisfaction in pregnant women. Our findings are consistent with both qualitative and quantitative studies reporting that pregnancy-related melasma is associated with a more negative body/face perception in women.^5,6^ In this context, pregnancy-related melasma should be considered not only a dermatological change but also an important condition that may affect women's body perception, and it should be addressed within a holistic approach in prenatal care.

The regression analysis revealed that education level, perceived income, history of melasma before and during previous pregnancies, family history of melasma, and skin type together explained 61% of the probability of melasma occurring during pregnancy. This demonstrates that melasma is not limited to pregnancy-specific physiological changes but constitutes a multifactorial condition resulting from the interaction of sociodemographic, obstetric, and genetic components, supporting the need for a holistic approach in risk assessment.

The results of this study suggest that high school and university graduation, as well as having an adequate income level, are factors that reduce the risk of melasma. Specifically, the probability of developing melasma was 5.26-fold lower in high school graduates and 7.09-fold lower in university graduates; also, it was 3.32-fold lower in pregnant women whose income equaled their expenses. This can be explained by the determining role of education and economic status in the health behaviors of individuals. The literature also reports that low income and education levels,^26^ socioeconomic status, and living conditions^27^ may be associated with the development of melasma. Women with low income and education levels may have a higher risk of melasma because of an increased likelihood of working outdoors and prolonged unprotected sun exposure, as well as limited access to dermatological care and treatment.^2,26^ Every ten hours spent outdoors per week increases the risk of pregnancy-related melasma by 27%.^28^ However, individuals with higher socioeconomic status have better access to dermatological care services and photoprotective products, which strengthens preventive health behaviors and potentially reduce the development of melasma.^27^ These results suggest that education and income levels exert protective effects, not only indirectly but also through behavioral mechanisms.

The history of melasma before pregnancy, during previous pregnancies, and familial predisposition significantly increased the risk of melasma in this study. The risk was 9.5-fold higher in those with a history of melasma before pregnancy, 14.16-fold higher in those who experienced melasma in a previous pregnancy, and 3.8-fold higher in those with a family history clearly, clearly demonstrating the recurrent nature of melasma and the role of individual/genetic predisposition. Another study conducted in Türkiye reported that the risk was approximately 44-fold higher in women with a history of melasma in previous pregnancies, and that family history was also significantly associated with this increase.^12^ Similarly, other studies emphasize a strong association between family history and melasma^23,24,28–30^ highlighting that genetic factors are among the important determinants.^23,29^ Based on our findings and the available literature, pregnant women with an individual or family history of melasma should be considered a high-risk group.

In the present study, Fitzpatrick skin type IV (brown/dark skin) was found to be the strongest risk factor for pregnancy-related melasma, with the probability of melasma being 12.36-fold higher in this group compared with those with skin type II (light skin). The literature also indicates that having a dark skin type (Fitzpatrick skin types III, IV, and V) is a significant risk factor for the development of melasma and for the spread of existing lesions.^2,23,31^ Melasma is rarely detected in extreme skin types (Fitzpatrick skin types I and VI) because of the limited pigmentation response, whereas it is frequently observed in intermediate skin types (Fitzpatrick skin types III, IV, and V). Melasma is more common in individuals with moderate skin types because of their more pronounced and variable melanin production responses when exposed to triggering factors, such as ultraviolet radiation. Skin type I has a low tanning capacity and therefore cannot produce sufficient melanin, whereas in skin type VI, the existing pigmentation level is already high, which limits the production of additional pigment.^31^ Previous studies have reported that pregnancy, as well as having dark skin (skin type V) and lifelong sun exposure, are significant risk factors for melasma,^28,32^ The risk of severe melasma is approximately three-fold higher under these circumstances.^32^ In light of these findings, it is important that pregnant women with dark skin types be more closely evaluated for melasma development during prenatal checkups and provided with counseling on preventive measures.

## CONCLUSION

Pregnancy-related melasma is a common condition, affecting approximately 40% of pregnant women, and is associated with poorer body perception. Educational attainment and economic status were identified as protective factors, whereas personal history, genetic predisposition, and skin type emerged as important risk factors for its development. Accordingly, prenatal care should incorporate the assessment of physiological skin changes and body image perception. This should be accompanied by educational interventions aimed at increasing awareness of risk factors and appropriate counseling and supportive care.

## Data Availability

Data supporting the findings of this study are available upon reasonable request from the corresponding author, Rabia Atilla.
